# Clinical trials of 3D printing splints to avoid contracture development in burned children

**DOI:** 10.3906/sag-2104-170

**Published:** 2021-10-21

**Authors:** Atilla ŞENAYLI, Güven ÇANKAYA, Can İhsan ÖZTORUN, Hakan OFLAZ, Emrah ŞENEL

**Affiliations:** 1 Department of Pediatric Surgery, Faculty of Medicine, Yıldırım Beyazıt University, Ankara Turkey; 2 Department of Material Engineering, Faculty of Engineering, Yıldırım Beyazıt University, Ankara Turkey; 3 Department of Bioengineering, Faculty of Engineering, Gebze Technical University, Kocaeli Turkey

**Keywords:** Three dimension, printing, splint, children, burns, contracture

## Abstract

**Background/aim:**

We evaluated the feasibility of producing splints with 3D printer technology to prevent contractures in burned children in our clinical prospective study.

**Materials and methods:**

After approvals, children with burns greater than 2nd degree were included in the study. Age, sex, burn percentages, printing time, filament types, number of filament trials, splint suitability, patient and doctor comments, preclinical trials’ significances and financial impact were evaluated statistically.

**Results:**

Seventy-six trials were conducted on 18 patients. Fourteen of the patients were male and 4 are female. Average ages of boys and girls were 5 and 3, respectively. Burn percentage was 36.9 ± 13. Polylactic acid (PLAFlex), polyurethane (PolyFlex), semiflexible copolyester (nGenFlex), and thermoplastic polyurethane (TPU) were the main filaments that were used in the study. Printing time differed from 4 to 29 h according to body regions. Splints were suitable for 81.25% in upper extremity, for 66.7% in lower extremity, and for 100% in mouth. Burn percentage was significantly correlated with total number of filament (p = 0.049). Other statistical evaluations were insignificant.

**Conclusion:**

The 3D printer seems to be useful in children with burns. However, difficulties caused by some reasons like production must be overcome. By increasing clinical experience, this emerging custom-made technology may become standard, and documented problems can be solved.

## 1. Introduction

Burning is one of the most devastating diseases that can cause physical and social restriction [1]. There are 6.5 million patients suffering from this problem in the US alone [2]. The annual cost of dealing with these complications for the US is approximately 50 billion USD [2]. Although the magnitude of the effort against these difficulties are tremendously high, the biggest concern of physicians is that the patients do not adapt to splints during their treatment processes and especially during their physiotherapy, and they raise issues that will reduce their quality of life [2,3]. For this reason, investigators are trying to make splints more feasible by using technology, and new products are emerging [3–7]. The main purpose in these pursuits is to obtain a user-friendly, safe, compatible, and cost-effective product [2,4,7,8]. 

Three-dimensional (3D) printers have been used to produce therapeutic devices like prostheses and orthoses for about 20 years [4,5]. Among these, there are devices such as hearing aids, stethoscope, multisensor perception simulators, and lenses [4,9]. Nowadays, some products printed with 3D devices are put on the market for burn patients. For example, total body burn area calculations can be made with 3D printers, and there are studies to manufacture products against scar developments [10,11]. 3D software, especially in obese patients, is also used to measure the burned area [11]. Another engineering area in the burn is the production of flexible and stretchable materials. The construction of products such as facial padding for hypertrophic scar treatments attracts more attention [5]. 

Treatment trials for burned fingers in adult patients are being conducted to prevent contracture, but publications are limited [3,5,8,9,12]. There is even less literature on the use of 3D printers for contracture treatment in pediatric burn patients. Its application to prevent the development of joint movement restriction (contracture) in children or to treat contracture has not yet entered the clinic. For all these reasons, one of the interests of our study is whether this application can be put into clinical practice.

Our other research goal is to evaluate the cost effect for splints made by 3D printers [4]. In some studies, it is said that production can be done quickly and cheaply and can be preferred over classical manufacture [4]. 

## 2. Materials and methods

### 2.1. Approvals

The study was designed with the approval of the Ankara Children’s Health and Diseases Hematology-Oncology Training and Research hospital ethics committee (2016/020). The project’s financial support approval number of the Ministry of Health, General Directorate of Health Research was 31296424. Financial support of the study was 1,200,000 Turkish Liras (approx. 174.000 USD). The Project was planned for 2 years and then completed in 3 years, using the 1-year official extension permission. 

### 2.2. Inclusion criteria

Male and female patients under the age of 18 with a 2nd degree deep burn and/or 3rd degree burn and a risk of contracture development, namely, joint area and mouth burns were included in the study. The families were informed about the study, and their consents were obtained.

### 2.3. Laboratory design 

The study was conducted in Ankara Yıldırım Beyazıt University Medical Metrology Application and Research Center. A four-room laboratory was constructed. Two scanograms and a 3D printer were installed, and workstations used by a “3D printer operator” were established. Raise 3D N2 Plus branded 3D printer and Artec Eva 3B and Artec Space Spider 3D scanograms were used in the study. The computerized view and topography of the relevant body surfaces of the patients were taken with the scanograms. Afterwards the surfaces of the printed splints were glazed in the laboratory conditions. Thus, its roughness has been removed and its suitability has been evaluated.

### 2.4. Indicators

Effectiveness of 3D splint printing is evaluated with age, sex, region, and burn percentages of the patients. Printing time, printing materials (filaments), preclinical trials, clinical trials, patients’ comments (if she/he is old enough to express opinion), doctors comments, and conclusive results were evaluated. According to the opinions received, new productions were made. Since splint applicability to patients has been investigated, it was not prioritized to investigate whether the effect of preventing contracture occurred. 

### 2.5. Gumshields for microstomy 

Mouth splints are made to prevent mouth narrowing due to burns. The products made were circumferential and were 4.5–6 cm with a size difference of 0.5 cm. The reason for its various widths is to expand the stenosis developing in the mouth with intermittent and gradual use. 

### 2.6. Upper extremity (hands–arms)

Trials have shown differences in the form of single piece, multipiece, buttoned, variable thickness, constant thickness, and different pore openings with different materials. In addition, dressing materials with various properties (in terms of thickness and adhesiveness) between the burned surface and the splint have been tried.

### 2.7. Lower extremity (legs–feet)

Various shapes and thicknesses of splints were tried as mentioned in upper extremity.

### 2.8. Statistical analysis

Data analysis was performed using IBM SPSS Statistics version 17.0 software (IBM Corporation, Armonk, NY, US). Assumptions of normality for continuous variables were examined using the Shapiro–Wilk test. Continuous variables were shown as mean ± SD or median (min–max), where appropriate. Number of cases and percentages were used for categorical data. While the mean differences between groups were compared, Student’s t-test, otherwise the Mann–Whitney U test was applied for the comparisons of nonnormally distributed variables. Categorical data were analyzed using Fisher’s exact test. Spearman’s rank-order coefficients of correlation were calculated to determine the degrees of association between continuous variables. p < 0.05 was considered statistically significant.

### 2.9. Sample size

Sample size was not decided according to “sample size estimation”. As an antecedent study about clinical usage of 3D printing at childhood, we evaluated the availability of the theory. Therefore, according to inclusion and exclusion criteria, we evaluated sequential patients till the end of the financial support.

## 3. Results

A total of 76 trials were conducted on 43 body regions (7 mouths, 30 upper extremities, 6 lower extremities) of 18 patients. The mean ages of male and female patients are 5 and 3, respectively. Demography with detailed trials for localizations is shown in Table 1. To avoid complicated combinations of regions, splints were evaluated in more general anatomic definitions like upper-lower extremity and mouth to ease the results as seen in Table 2. Four different types of filaments were used for splint printing. These are PLAFlex, PolyFlex, nGen Flex, and TPU. Some combinations of filaments were also tried. Number of trials and related regions that the filaments were used were shown in Table 3a. One type of filament was mostly used for patients, but in some of them, more than one type was used to structure a righteous form. Numbers of different filaments were shown in Table 3b. Age, sex, and burn percentages were not significant according to number of splint regions, doctor comments, or conclusive results demonstrated in Table 4. Moreover, printing time and preclinical and clinical trials were not correlated with age and burn percentage. Total filament numbers according to burn percentage were found significant (p < 0.05) as shown in Table 5. Statistical evaluation of age, burn percentage, number of splint regions, total filament numbers, total printing time, and total preclinical and clinical trials were not significantly correlated with sex.

**Table 1:** The demography of the patients who were prepared with a 3D printer and the number of times the trials were performed. *Trials with gumshield. Gumshiled trials were not included in the total since they were not routinely planned.

Patient no	Age(year)	Sex	Left hand	Left arm	Right hand	Rightarm	Mouth	Right foot and leg	Left foot and leg	Burn %
1	12	M	2		2		2			51
2	8	M			3		2 + 3*			58
3	4	M	3		1		2			35
4	3	M					1			20
5	3	F	2		2					20
6	1	F						1	1	31
7	3	M	1		1		2 + 2*	3	3	27
8	4	M	1		2		2	2	2	45
9	2	M	2		2					50
10	9	M	2	2	2	2				44
11	3	F	2		2		2			44
12	5	M				2				40
13	1	M			3					10
14	11	M	3							35
15	2	F	1		1					39
16	16	M	1							30
17	1	M				1				30
18	6	M	1	1	1	1				55

**Table 2:** Demographic and clinical features of the patients.

	n = 18
Age (year)	3.5 (1–16)
Sex	
Male	14 (77.8%)
Female	4 (22.2%)
Burn percentage	36.9±13.0
Splint region	
Upper extremity	16 (88.9%)
Lower extremity	3 (16.7%)
Mouth	7 (38.9%)
Number of splint regions	
Only one region	12 (66.7%)
Two regions	4 (22.2%)
Three regions	2 (11.1%)

**Table 3a:** Filament types according to the localization. Polyflex (polyurethane); polylactic acid (PLAflex); thermoplastic polyurethane (TPU), semiflexible copolyester (nGenflex).

	Filament trials (number)	Percentage
Upper extremity	30	100.0
PLAFlex	11	34.4
PolyFlex	11	34.4
nGenFlex	2	6.5
PolyFlex/nGenFlex	2	6.5
TPU	1	3.3
nGenFlex/TPU	1	3.3
TPU/PLAFlex	1	3.3
TPU/PolyFlex	1	3.3
Lower Extremity	6	100.0
PolyFlex/GenFlex	2	33.3
PolyFlex/PLAFlex	2	33.3
TPU	2	33.3
Mouth	7	100.0
PolyFlex	7	100.0

**Table 3b:** Frequency distribution of filament numbers according to the localization.

	Patient numbers	Percentage
Upper extremity	16	100.0
1 filament	6	37.5
2 filaments	8	50.0
4 filaments	2	12.5
Lower extremity	3	100.0
2 filaments	3	100.0
Mouth	7	100.0
1 filament	7	100.0
Total	18	100.0
1 filament	6	33.3
2 filaments	5	27.8
3 filaments	3	16.7
4 filaments	2	11.1
5 filaments	2	11.1

**Table 4:** Demographic and clinical features of the patients according to region numbers, doctor comment and conclusive result

	Splint in one region(n = 12)	Splint in more than one region (n = 6)	p-value
Age (year)	3 (1–16)	4 (3–12)	0.385†
Sex			>0.999‡
Male	9 (75.0%)	5 (83.3%)	
Female	3 (25.0%)	1 (16.7%)	
Burn Percentage	33.7±13.0	43.3±11.1	0.140¶
	Suitable according to doctor comment (n = 8)	Not suitable according to doctor comment (n = 10)	p-value
Age (year)	3 (1–16)	4 (1–12)	0.965†
Sex			0.275‡
Male	5 (62.5%)	9 (90.0%)	
Female	3 (37.5%)	1 (10.0%)	
Burn percentage	32.2±12.4	38.2±13.9	0.645¶
	Conclusive resultpartial successful (n=6)	Conclusive resultsuccessful (n = 12)	p-value
Age (year)	3 (1–12)	3.5 (1–16)	0.553†
Sex			>0.999‡
Male	5 (83.3%)	9 (75.0%)	
Female	1 (16.7%)	3 (25.0%)	
Burn percentage	39.2±17.6	35.8±10.7	0.613¶

†Mann–Whitney U test, ‡Fisher’s exact test, ¶Student’s t-test.

### 3.1. Gumshield trials (microstomy) 

**Figure 1:**
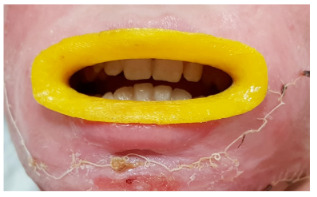
Gumshield application example. Circumferential type was preferred for intermittent therapies.

Gumshields were printed in approximately 4 h (2–4) as seen in Figure 1. After 4 preclinical trials in median (2–10), they were used in clinics. All of them were printed successfully as defined in Table 6. However, one of them defined pain. As a conclusive result, they were found useful. Two patients developed teeth grinding problem due to psychological reasons after a while. In order to prevent damage to the teeth, a total of 5 gumshield tests have been made with a 3D printer. Gumshield trials were not included in the total evaluation figures as they were not in routine planning.

**Table 5:** Correlation coefficients and significance levels according to filament numbers with age and burn percentage.

	Age		Burn percentage	
	Correlationcoefficient	p-value †	Correlationcoefficient	p-value †
Number of region for splints	0.192	0.444	0.310	0.210
Total filament numbers	0.157	0.533	0.471	0.049
Total printing time	0.029	0.909	0.283	0.255
Total preclinical trial	0.216	0.405	0.353	0.164
Total clinical trial	0.237	0.344	0.256	0.305

†Spearman’s rank order correlation analysis.

**Table 6:** Clinical findings according to localizations.

	Upper extremity(n = 16)	Lower extremity(n = 3)	Mouth(n = 7)
Printing time (hour)	9 (6–13)	18 (15–29)	4 (2–4)
Preclinical trial	2 (1–4)	2 (2–2)	4 (2–10)
Clinical trial	2 (1–3)	2 (1–3)	2 (1–5)
Trial results			
Suitable	13 (81.25%)	-	7 (100.0%)
Thickness problem	2 (12.50%)	-	-
Unsuitable for dressing	1 (6.25%)	-	-
Cutting necessity	-	1 (33.3%)	-
Model change	-	2 (66.7%)	-
Patient comment			
Suitable	4 (25.0%)	-	-
Too young to comment	9 (56.25%)	3 (100.0%)	2 (28.6%)
Pain	1 (6.25%)	-	1 (14.3%)
Unconscious	1 (6.25%)	-	-
Pain in joint	1 (6.25%)	-	-
Not matter	-	-	4 (57.1%)
Doctor comment			
Suitable	8 (50.0%)	-	6 (85.7%)
Not Suitable	8 (50.0%)	3 (100.0%)	1 (14.3%)
Conclusive result			
Revision	3 (18.75%)	-	-
Partial Success	2 (12.50%)	1 (33.3%)	-
Suitable	11 (68.75%)	2 (66.7%)	7 (100.0%)

### 3.2. Upper extremity

Splints for upper extremity were printed 9 h in median (6–13). After 2 preclinical trials, they were used in clinics as shown in Table 6. In this group, suitability is found less (81.25%). As a conclusive result, 11 splints were found useful as shown in Table 6. During the scanning procedure, it was realized that burned surfaces could not be scanned as well as healthy surfaces as seen in Figures 2a and 2b. Moreover, after scanning, some other measurements must be taken into account to sustain the dressing management and preventive effect of splints as shown in Figures 3a and 3b.

**Figure 2a:**
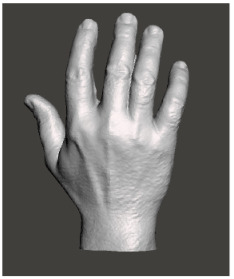
Scanogram of the back view of one of the investigators (G.Ç.) hand. Note that it is comprehended distinctly compared to burn patients.

**Figure 2b:**
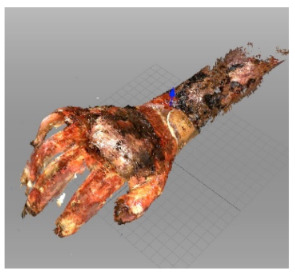
In the comparison of the burned patient and Figure 2a, it can be easily seen how different results can be obtained in the same device. The perception of depth is damaged.

**Figure 3a:**
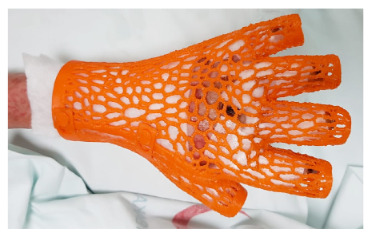
Gap work between the burned surface area and splint: finger adjustment trial.

**Figure 3b:**
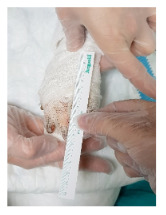
Hand distance measurement considerations.

### 3.3. Lower extremity

**Figure 4:**
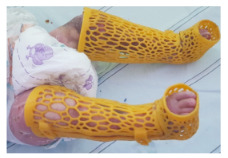
Foot and leg splint (without intermediate dressing).

 This group was the smallest one in number as shown in Table 6. Moreover, patients in this group were younger. We, therefore, presume that unsuccessful results of printing are because of these factors at first attempt, as shown in Table 6. After revised trials, 2 of the splints in 3 trials were suitable for usage as shown in Figure 4.

### 3.4. Costs

All the funding was used in the project. 3D printer and scanogram cost about 15,000 USD. The cost of the supplies used was between 1.6 and 5 USD. These amounts were deemed appropriate for routine use, but it was thought that the total cost of practical consumption would increase as a result of adding other facts that increase the cost like labor cost or depreciation.

## 4. Discussion 

One of the reasons for applying splint in burn patients is to prevent joint restriction, namely contracture, in 2nd degree deep or 3rd degree burns. In fact, the use of splints is also beneficial to eliminate the psychological, social, and economic problems that may develop in the long run by preventing the formation of joint restriction. 

The main material of the classical splint is plaster. Thermoplastic products are also used. However, there are several challenges with producing splint in this way: positioning difficulties, contamination, and using tools such as rulers or photographs [2]. After all, especially in children, a splint may not be prepared properly for the limb. For these reasons, it is natural to be in search of new methods and devices by using technology.

The use of 3D printers is a promising development. It has been determined that three-dimensional printers can be used in many ways in burn patients. For instance, by measuring total body surface area, fluid and metabolic treatments can be planned for the patient [11]. Another example is its use for dressings or treatments made with 3D software produced meshes [11]. Trials of splint production by using 3D printers for contractures in various parts of the body are ongoing. The attractive aspects of producing a splint with 3D are comfort and adequacy, appropriate ventilation of the tissue/skin, lightweight, and aesthetic features [4]. In our study, attempts were made to test these characteristics of 3D splints against mouth stenosis (microstomy), and also the upper-lower extremity joint burns contractures.

### 4.1. Gumshield (microstomy)

If care is not taken during the scar formation process in facial burns, microstomy may develop with the narrowing of the oral commissure due to scar contractions [13–15]. The main factor of this situation is the inability of orbicularis oris muscle to resist the developing scar tissue [13]. Ordinary opening of the mouth is 40–50 mm on average [13]. If this average oral opening is 25–35 mm, it can be considered functional, but if it is 10–24 mm, a microstomy should be identified [13]. In this case, surgery is usually performed for treatment [13,16]. Producing a splint using a 3D printer, applying pressure and trying to eliminate the hypertrophic scar effects is an emerging therapy as another method [8,17]. There are a limited number of studies in the literature on this subject. Some of these publications were made on adult patients and the splints were mostly designed as handheld [15]. It is seen that the mouth splints produced in these literature are of three different types in terms of shape: horizontal, vertical, or circumferential [14,15]. They were divided into two types of use: continuous use or intermittent use [15]. It is stated that the use of mouth splints can be beneficial; however, problems may occur [14–16]. Problems such as a mood of limitation in daily activity, rejection of splint, nutritional problem, dental hygiene, speech articulation, aesthetic concerns, psychological dissatisfaction, respiratory problems, and aspiration incidences have been reported. These are important limitations of practical use [14,15,17]. Noncompliance of the patients to gumshields was the most cited problem [15]. Therefore, it is thought that the application of removable dynamic splints will be more appropriate and it has been stated that the pressure can be adjusted [14]. Despite all the precautions, there still may be a pressure problem [15]. Usage periods of mouth splints are recommended as 3–6 months [14]. 

In our study, our preference was made in various sizes and by producing a circumferential mouth splint to be suitable for intermittent use for the expansion of the commissure. Meanwhile, it was experienced whether the patient tolerated and would benefit from it. At first, all but one tried the gumshields, but after a while, they did not want a splint in their mouth. In fact, after a short while, the splints could not be used as patients resist opening their mouths. Therefore, systematic usage could not be achieved. There was no problem with the production of the splint. It has been spotted that the production time and cost of mouth splints are also not a problem. We concluded that the strategies on patient compliance must be developed. However, it is not an aim of this study.

### 4.2. Upper extremity (hands–arms)

Hand burns are the most frequently encountered burns among the parts of the human body [5,12]. It can be seen with damage such as contractures, hypertrophic scarring, and peripheral nerve damage [12]. Pressure therapy and/or silicone gel sheets are used for the treatment of hypertrophic scars in this region [5]. Although splint applications on the upper extremities have developed over time, the complex geometric structure of the human body always contains the possibility of causing troubles [5]. If this complex part of the body is not treated carefully, the development of hypertrophic scars may be confronted [5]. In addition, more applications had to be made for special problems in the fingers [12]. For all these reasons, improvement of the orthotic applications is very important for achieving treatment success [12]. 

There is a huge amount of studies in the literature on wrist splints reporting that individual products can be produced in various ways and methods such as thermoplastic technique can be used for this purpose [3,12]. As a promising technique, curiosity in hand splint production with the use of 3D printers is increasing [12]. Advantages such as being cheap are also an attractive reason for 3D printing investigations [12]. 

The biggest benefits of splints made with three-dimensional printers are that the measurements are very accurate and therefore cause little unsuitability problems [8]. However, difficulties such as the hardness-softness relationship at the skin contact points, and the characteristics of lattice formations may be disturbing [3]. In a study conducted by Paterson et al. in the UK, splints were tried on 10 healthy volunteers [3]. However, producing splints for unhealthy people might be more complicated. It has been reported that among the biggest problems seen for wrist splints are keeping it clean and dry, adjusting, removing and reattaching, and the problems in the process of resolving the edema [3]. Some studies have also reported that the patients do not feel comfortable and have limited benefit from splints [3,9]. In addition, problems such as itching, sweating, contact dermatitis, and improper configuration can lead to inadequate treatment [5,6]. In some other studies on fingers, a similar problem was found in adult patients [8,9,12]. 

Problems in our study are mostly similar. Although the hand-arm splints were mostly produced in accordance with the patients in our study, the number of trials was higher than the other body surfaces due to differences such as model and appearance changes, being single-/multipiece, and buttoned or button-free. The main problem observed in our patients is related to the patients’ tolerance of splints. However, these problems are not disappointing. Compliance with the use of a splint was observed in one of our patients, and the risk of contracture development was eliminated in this patient. Based on this example, we can say that with the elimination of the existing problems, the splints produced with the 3D printer to be used in the future will be at least as effective as splints produced using classical plaster.

### 4.3. Lower extremity (legs-feet)

After foot burns, the probability of advanced deformity that causes posture and gait difficulties is between 5% and 7% [18]. When there is a problem in the lower extremities, irregular and asymmetrical foot movement patterns that are related to each other often occur due to kinematic reasons [1,19]. Due to the synergy that develops with this disorder, problems occur in the central nervous system and an increase in muscle tone [1]. It also initiates processes that will cause stress disorders in the patient [19]. In a study conducted with adults, the rate of burn patients with a decrease in “range of motion” (ROM) was found to be 18.5% [20]. In burns, this situation occurs with contractures [21]. These contractures must be released to ensure normal movement in the extremities [21]. The most common treatment is contracture release by scar incision and skin grafting [19). Advanced treatments and rehabilitation may be required along with surgical procedures [20]. 

Orthoses are produced for lower extremity diseases against foot pain and gait disturbance, but these diseases are not burns [22]. Moreover, it was seen that only in 1 article that was cited in a Cochrane study accomplished in 2008, 3D printer was used to produce orthosis [23]. In the following years, investigations generally continued in the form of under-research of the 3D printer [24]. In some studies, it was determined that examinations were made with 3D motion analysis [20]. 

Our trials of 3D splint printing are one of the rare studies on childhood lower extremity burns. It is not successful in long-term usage for contracture prevention but it is encouraging for carrying on testing. Considering our results of splint production with 3D printing, splints produced for lower extremities have failed, as in other body parts, with ineffectiveness, discomfort, incongruity, puffy posture, poor aesthetics, skin irritation, blistering, or tightness [24]. There was no skin irritation or blister formation in our patients, but unfortunately, all the other problems were seen. Despite the rarity of 3D printer application data on lower extremity burns, it is stated that treatment programs can be performed with increasing information [20,21]. By studying with a 3D technology, motion limitation of patients and the benefits of therapy can be analyzed [21]. 

### 4.4. Scanogram 

The technical materials used in our study were also evaluated. For the production of splints to be used in pediatric burn patients, it is understood that the topography of the body area should be extracted in more detail when compared with normal body areas. In other words, to achieve higher efficiency, it is necessary to use scanograms with higher resolution and faster scanning. The reason of the need for being fast is about children’s incompatibility to stand still. A similar opinion was expressed in another study, and it was stated that scanograms could make measurement inadequate [17]. In our study, as shown by comparing Figures 2a and 2b, although we obtained a very good topographic result in one of our researchers (G.Ç.), the same quality could not be achieved in the patients. Alternatively, it may be thought that topographic evaluation can be done with methods other than the use of scanograms. One of these methods is computed tomography defined as computer-aided design (CAD) [3]. However, this process also has the problem of artifact formation and is a disadvantage for topography extraction [6]. It is obvious that the procedure cannot be performed at the bedside with CAD. It can also cause data loss, cost increase, and patient incompatibility [3]. 

There are two important reasons for the effectiveness of the scanogram process for our study. The first reason is that a space suitable for wound dressing should be left between the splint and the burn area, because if it is desired to eliminate the risk of contracture development by using a splint, a splint will naturally be worn during burn treatment. At the same time, necessary treatments and dressings must be continued. Otherwise, the contracture development process will advance, because of all these facts, it is important that the scanogram must show the burned surface exactly and clearly. It must define the thinning-thickening areas on the burned surface.

The second reason why the scanogram resolution should be very good is to understand how to place the splints especially between the fingers. One of the most important complications of hand burns is finger contracture. Therefore, it is necessary to make very precise adjustment to protect the fingers against contracture; consequently, the resolution of the scanogram examinations should be very good. 

### 4.5. 3D printer

The size of the 3D printer, the software used, and the price of the printer are important [4]. The price of a printer that can be used by a beginner can be 200–300 USD [4,8]. We think that it is very difficult to produce splints like ours with beginner type. According to our experience, a higher-capacity printer should be used, but professional and even industrial 3D printers are quite expensive [4]. We used a professional printer in our study. The 3D printer we used in our study was a professional type printer and its approximate cost was 8500 USD. However, we realized that more successful results could be achieved if the top-level industrial type was used. This means a more expensive 3D printer. The price of a printer at this level is between 20,000 and 100,000 USD [4]. If an expensive printer is purchased, filaments will also be more expensive [4]. 

The properties of the filament fibers used for splint production are also important when producing splint with a 3D printer. The material to be used must have sufficient strength and flexibility [4]. In order to achieve this, variable splint thickness can be programmed in the same splint. At the beginning of the study, the production of constant thickness material was tried. When the constant thickness application was observed to be insufficient, varying thicknesses were tried in different parts of the splint. The parts that come to the joint areas and fingers are made thicker. However, despite this, bending and fracture occurred in both hand and foot splints due to patients’ strain. Depending on the patient’s incompatibility and strength, the splint should be produced in a way that the material to be used can resist sustainably against developing contracture. There are studies reporting similar problems [3,4,25]. Conversely, some studies indicate that the efficacy is good, but these studies are mostly performed in adults and are mostly due to fractures [6]. 

One of the most curious issues in splint production with 3D printers is cost [3]. The prices of the consumables used in splint production in our study are between 1.6 and 5 USD. This figure is an affordable price per unit. In the study by Kadioğlu et al., the splints produced for the hand and/or fingers were produced at a cost of 5–10 USD and were produced from PLA [6]. It was stated in the same study that prices could be reduced [6]. In addition, as stated in another study, orthosis were produced in 1 h in the partial splint study related to the fingers and average cost was 1 USD [12]. It was also stated that there was no amount of production that could be statistically compared [12]. In the splint study carried out on the face, the total transaction amount per person was expressed as 625 Great Britain Pound (GBP), which is quite high compared to the standards of our country [17]. It is still possible to understand why the prices are so high. 3D production is different from conventional applications, and requires a special laboratory environment, 3D printer, scanogram, retouching workshop, and the employment of technical personnel with training and skills to use the devices. These necessities may explain the increase in per capita price. It has been noted that especially the software and machines increase the cost [25]. Additional costs are reported to be between 167 USD and 4000 USD [25]. In a study performed in the UK, the maximum cost to establish such a system is expressed £ 700,000 [3]. In another study, it was emphasized that the time and cost spent with the use of 3D printers may not be satisfactory [8]. Of course, high prices per patient are a disadvantage for 3D products [25].

Production time should be considered in material production with 3D printer [25]. In some products, this period is stated to be between 10 h and 3 weeks [25]. In our study, the topography planning of the cases, obtaining scanogram results, and the retouching time were variable and generally long. Due to the fact that the printer we use can print some splints for a long time up to 24 h, it can be expected to have a cost-increasing effect when the number of productions is low. When other expenses are taken into account, it is inevitable that few productions will increase the cost. 

Another problem in the literature is the efficiency comparison of classical splints and splints made by 3D printers, as in our study. There are articles stating that products manufactured with 3D printers do not have superiority compared to conventional production [8]. This may be a future discussion of authors, but nowadays a large database that allows statistical evaluation has not yet been established. Our study is one of the pioneers.

In our study, we showed that splint could be produced in pediatric burn patients by printing with a 3D printer. We observed that this technique is a promising one for burn patients, and further investigations must be done with more funding.

## 5. Conclusion

In the pediatric burn patients group, this is the first time a comprehensive 3D printed splint trial has been made. Due to the nature of this very special patient group, it was essential to deal with many variables. It has been understood that the 3D printer can be used safely in children with burn problems. Concrete data on laboratory conditions, production settings and material properties have been formed, as we presented in this article. In clinical practice, by increasing the number of patients, algorithms can be developed for the method of use and the mentioned problems can be overcome. This project can offer innovation to clinical practice if financial support continues.

## Informed consent

The study was designed with the approval of the Ankara Children’s Health and Diseases Hematology-Oncology Training and Research hospital ethics committee (2016/020). The project financial support approval number of the Ministry of Health, General Directorate of Health Research was 31296424.
